# Inhibited Differentiation and Growth of Myocyte Associated With Sarcopenia: The Key Role of the lncRNA A430093F15Rik/microRNA‐337‐3p/Fam168a Pathway

**DOI:** 10.1111/jcmm.71133

**Published:** 2026-04-16

**Authors:** Qing Fang, Jianwei Huang, Dongping Huang, Zhenyi Jia

**Affiliations:** ^1^ Department of Clinical Nutrition Putuo People's Hospital, School of Medicine, Tongji University Shanghai People's Republic of China; ^2^ Department of General Surgery Putuo People's Hospital, School of Medicine, Tongji University Shanghai People's Republic of China; ^3^ Department of General Surgery Shanghai Sixth People's Hospital Shanghai People's Republic of China

**Keywords:** A430093F15Rik, ceRNA, Fam168a, miR‐337‐3p, sarcopenia

## Abstract

Sarcopenia is a muscle disorder characterized by progressive loss of muscle mass, strength and function with ageing. Non‐coding RNAs have been reported to be involved in the progression of sarcopenia. The current study aimed to investigate the pathogenesis of sarcopenia. Based on the bioinformatics analyses and RT‐qPCR validation, the lncRNA A430093F15Rik was selected as the potential target involved in sarcopenia progression. Its expression level was up‐regulated with ageing in mice but down‐regulated with myogenesis in C2C12 cells. Modulating A430093F15Rik showed that the inhibition of the lncRNA contributed to the attenuation of sarcopenia such as increased cell viability and enhanced myogenesis, while the overexpression promoted disease progression. The downstream effector of A430093F15Rik, miR‐337‐3p, showed opposite function to the lncRNA, while Fam168a showed similar effects. Moreover, modulating both factors also confirmed their distinct roles during sarcopenia progression. The dual luciferase and RNA pulldown assays then verified the direct binding between A430093F15Rik and miR‐337‐3p, and miR‐337‐3p and Fam168a, representing a ceRNA regulatory mechanism between A430093F15Rik, miR‐337‐3p and Fam168a. The current study identified a novel lncRNA, A430093F15Rik, that is involved in the progression of sarcopenia by acting as a competitive endogenous RNA (ceRNA) to sponge miR‐337‐3p and regulate the expression of Fam168a.

## 1 Introduction

Sarcopenia is one of the predominant factors contributing to the formation of muscle atrophy in *Homo sapiens* , which was first described as “loss of flesh” by Rosenberg in 2011 [1]. The disorder is characterized by symptoms including progressive loss of muscle mass, strength and function with age [2] and will result in multiple negative outcomes such as increased risk of frailty, decline in physical function, falls and higher mortality rates [3]. It is well established that sarcopenia is the major cause of disability and loss of independence in the elder population. Worse still, sarcopenia can also cause manifestations in the younger population under certain circumstances, such as bed rest, sedentary lifestyle, severe malnutrition or chronic diseases including cancer and chronic heart failure [4], which has placed a great burden on the public health system and caregivers. Although the harms associated with sarcopenia have been universally reported, effective treatment strategies for the disorder have not been established. Currently, both non‐pharmacological and pharmacological strategies are recommended to be integrated into the treatment approach, but the applicability may be limited for certain populations. For instance, resistance exercise that is well recognized to benefit sarcopenia patients cannot be applied to cases with physical disabilities. Thus, it is crucial to explore alternative strategies that offer effective interventions on the impairments associated with sarcopenia.

Emerging evidence demonstrates that the initiation of age‐related sarcopenia is induced by signalling pathways, including transforming growth factor beta (TGF‐β), bone morphogenetic protein (BMP) and insulin‐like growth factor 1 (IGF‐1), etc. [5, 6, 7, 8]. The specific modulation of these pathways contributes to the attenuation of sarcopenia [9, 10, 11]. Therefore, it is reasonable to explore novel targets that mediate the progression of the disorder, which may facilitate the development of treatment strategies against sarcopenia. Non‐coding RNAs, including microRNAs (miRs) and long non‐coding RNAs (lncRNAs), are involved in multiple biological processes and disorders [12, 13]. The former type is small non‐coding RNAs that are 21–25 nucleotides (nts) in length, and the latter is more than 200 nts in length. Both types can regulate gene expression in multiple ways: miRs recognize specific target mRNAs and lead to translational repression or mRNA degradation [14], and lncRNA can modulate gene expression via epigenetic, transcriptional, post‐transcriptional and translational regulation [15]. Regarding the role of non‐coding RNAs in the skeletal muscle development, previous studies have shown that they are involved in the skeletal muscle regeneration and myogenesis [16, 17]. Moreover, several miRs and lncRNAs have been implicated in skeletal muscle diseases, including sarcopenia. For instance, miR‐206 is overexpressed in the diaphragm but not the hindlimb muscle of mouse model [18], and the DMD locus harbours multiple lncRNAs which control the expression of dystrophin [19]. It is also confirmed that the differential expression of lncRNAs with ageing exacerbates the symptoms of sarcopenia [20]. Thus, a comprehensive exploration of the expression profile of the non‐coding RNAs in sarcopenia patients or animal models may provide valuable information for identifying novel targets for the treatment of the disorder.

In the current study, we first employed datasets archived in Gene Expression Omnibus (GEO) to identify potential non‐coding RNAs involved in the pathogenesis of sarcopenia. Based on a series of bioinformatics analyses and RT‐qPCR validation, lncRNA A430093F15Rik and miR‐337‐3p were selected in the current study, and the expression levels of the two non‐coding RNAs were modulated both in vitro and in vivo to verify their key roles in the development of sarcopenia.

## 2 Methods

### 2.1 Data Download and Bioinformatics Analyses

Sequencing data regarding miRs and mRNA/lncRNA related to the initiation of sarcopenia were retrieved from two GEO datasets, GSE55164 and GSE55163. The differentially expressed molecules were identified using limma package version 3.10.3 [21], with the threshold that *P* value was smaller than 0.05 and |logFC| > 0.5. The co‐expression of lncRNA and mRNA was analysed using clusterProfiler package version 3.8.1 [22]. The potential interaction between lncRNA, miR and downstream targets was analysed using miRWalk version 3.0 [23] and Miranda version 3.3a. The ceRNA network was then built using Cytoscape version 4.0 [24]. The selected lncRNAs and miRs were then subjected to RT‐qPCR validation using musculus gastrocnemius tissues collected from mice of different ages. Based on a series of assays, lncRNA A430093F15Rik, miR‐337‐3p and Fam168a were selected for the subsequent explorations.

### 2.2 Animals and Grouping

Male C57BL/6J mice (eight weeks old) were purchased from Changsheng Biotech Inc. (Liaoning, China) and were kept under routine conditions for 24 months. Female mice were not included because accumulating evidence indicates that recent reports show that aged male C57BL/6J mice display a 9%–22% incidence of sarcopenia, while aged female mice exhibit far fewer confirmed cases [25]. The musculus gastrocnemius tissues were collected from mice of two, six and 24 months old, respectively, and the ratio of musculus gastrocnemius weight to body weight was recorded. All procedures were conducted in accordance with the National Research Council's Guide for the Care and Use of Laboratory Animals, and the protocol was approved by the Institutional Committee for the Care and Use of Laboratory Animals (DWSY2023‐0178, Shanghai Sixth People's Hospital, China).

To assess the role of A430093F15Rik modulation in the progression of sarcopenia, 36 mice were randomly divided into six groups (six for each group): A, six‐month‐old mice; B, 24‐month‐old mice; C, 24‐month‐old mice with A430093F15Rik inhibition via tail injection of A430093F15Rik shRNA (5′‐TGCTGATGCTGAGGGAAATAT); D, six‐month‐old mice subjected to vehicle injection; E, six‐month‐old mice subjected to DEX treatment (25 μg/g) for the induction of sarcopenia; F, six‐month‐old mice subjected to DEX treatment (25 μg/g) for the induction of sarcopenia with A430093F15Rik inhibition via tail injection of A430093F15Rik shRNA. Upon completion of the assay, mice were sacrificed with overdose pentobarbital sodium, and musculus gastrocnemius tissues were collected for subsequent assays.

### 2.3 Real Time Quantitative PCR (RT‐qPCR)

To determine the expression level of target genes, total RNA or nuclear RNA was extracted for different samples using the TRIzol solution (15,596,018, Thermo, China). Total RNA was treated with gDNA Eraser (Takara RR047Q) to remove genomic DNA prior to reverse transcription. For quality control, no‐RT controls were included during assay optimization to confirm the absence of genomic DNA contamination. cDNA templates were generated using a high‐capacity cDNA reverse transcription kit (RR047Q, TaKaRa) and 1 μg RNA was used as the template for qPCR amplification with qPCR kit (RR420Q, TaKaRa, China) using ExicyclerTM 96 (BIONEER, South Korea). The primer information is shown in Table S1 and the relative expression levels of target genes were calculated according to the formula of 2^₋△△ct^.

### 2.4 Western Blotting

Protein samples were prepared via lysis of cells using a water bath at 100°C and the protein content was determined by a modified BCA protein assay. Subsequently, the samples (15 ~ 20 μL) were subjected to sodium dodecyl sulphate‐polyacrylamide gel electrophoresis (SDS‐PAGE) using an Electrophoresis and Blotting Apparatus (Bio‐Rad), and protein products were then transferred to nitrocellulose membranes. After being blocked with 3.0% BSA for 1.5 h at room temperature, the membrane was incubated with primary antibodies for Fam168a, MyoD, MyoG, Mef2c, Myf5, MyhC, Cyclin E, Cyclin D1, PCNA and β‐actin overnight at 4°C. Protein bands were subsequently developed using the ECL reagent (36208ES76, Yeasen, China) and the results were captured using a visualizer (Las‐4000, GE). The relative protein expression level was calculated with β‐actin as the internal reference.

### 2.5 H&E Staining and Immunofluorescent Assay

H&E staining was used to observe the histological changes in muscle tissues: briefly, tissues were perfused‐fixed with 4% formaldehyde using Bouin solution. Once dehydrated, they were vitrified in dimethylbenzene and treated with varying alcohol concentrations. Then, samples were embedded in paraffin, sectioned and stained with H&E to reveal the results under a microscope at 100× magnification. The cross sectional area of tissues was also recorded.

Muscle tissues were blocked for 15 min with 10% goat serum after being permeabilized for 30 min with 0.5% Triton X‐100. Following this, tissues were allowed to come to room temperature for five minutes before being stained with MyHC and laminin. A fluorescent microscope operating at 100× magnification was used to image the distribution of positive cells.

### 2.6 Cell Grouping

To further explore the interaction between lncRNA A430093F15Rik, miR‐337‐3p and Fam168a, the levels of these molecules were modulated in C2C12 cells (iCell‐m013, Icell, China). Firstly, C2C12 cells were cultured in DMEM medium supplemented with 10% FBS for seven days, and cells were collected at the 1st, 4th and 7th days for detecting the expression levels of A430093F15Rik and other myogenesis indicators.

Then, the cells were divided into different groups by modulating the expression level of A430093F15Rik: A, C2C12 cells; B, C2C12 cells transfected with NC vector; C, C2C12 cells transfected with A430093F15Rik expression vector; D, C2C12 cells transfected with NC shRNA; E, C2C12 cells transfected with A430093F15Rik shRNA.

The function of miR‐337‐3p was explored by inducing the expression of miR‐337‐3p using mimics: A, C2C12 cells transfected with NC mimics; B, C2C12 cells transfected with miR‐337‐3p mimics.

The interaction between A430093F15Rik and miR‐337‐3p was validated using a rescue strategy by dividing C2C12 cells into groups: A, C2C12 cells; B, C2C12 cells transfected with A430093F15Rik expression vector; C, C2C12 cells transfected with A430093F15Rik expression vector and NC mimics; D, C2C12 cells transfected with A430093F15Rik expression vector and miR‐337‐3p mimics; E, C2C12 cells transfected with mutant A430093F15Rik expression vector and miR‐337‐3p mimics.

The role of Fam168a was then explored by first dividing C2C12 cells into groups: A, C2C12 cells; B, C2C12 cells transfected with NC shRNA; C, C2C12 cells transfected with Fam168a shRNA; D, C2C12 cells transfected with empty vector; E C2C12 cells transfected with Fam168a vector. Then, a rescue strategy was also employed to confirm the interaction between A430093F15Rik, miR‐337‐3p and Fam168a: A, C2C12 cells; B, C2C12 cells transfected with A430093F15Rik expression vector; C, C2C12 cells transfected with A430093F15Rik expression vector and NC shRNA; D, C2C12 cells transfected with A430093F15Rik expression vector and Fam168a shRNA; E, C2C12 cells transfected with miR‐337‐3p mimics and empty vector; F, C2C12 cells transfected with miR‐337‐3p mimics and Fam168a expression vector.

Upon completion of different treatments, cell samples were collected and subjected to subsequent corresponding assays.

### 2.7 Fluorescence In Situ Hybridization (FISH)

The subcellular location of A430093F15Rik was detected using FISH: briefly, cells were fixed in 4% paraformaldehyde, treated with protease K, glycine and acetylation reagent, incubated with hybridization solution and probe‐labelled A430093F15Rik overnight. Afterward, cell nuclei were stained with DAPI. The pictures were captured using a laser scanning confocal microscope.

### 2.8 Cell Viability Detection

Cell viability was measured via staining with CMFDA: briefly, confluent cell layers were incubated with CMFDA for 45 min and then fixed with 10% paraformaldehyde. The relative cell viability was calculated using fluorescent density as the indicator, detected under a fluorescent microscope. For CCK‐8 assays, C2C12 cells were incubated with 5 mg/mL CCK‐8 for another 4 h after treatment. Cell viability was determined by measuring OD_490_ values using a microplate reader (ELX‐800, BIOTEK, USA).

### 2.9 RNA Pulldown

Biotinylated A430093F15Rik (Biotin‐sense) and its negative control (Biotin‐antisense) were generated by Sango Biotech (Shanghai, China). Cells were lysed and incubated with streptavidin‐coated magnetic beads containing Bio‐A430093F15Rik or Bio‐NC. Following elution, the levels of A430093F15Rik and miR‐337‐3p were detected using RT‐qPCR detection.

### 2.10 Dual Luciferase Assay

The interaction between A430093F15Rik, miR‐337‐3p and 3’UTR of the Fam168a gene was detected using a dual luciferase assay. Vectors carrying the wild‐type (WT) and the mutant type (MUT) of A430093F15Rik or 3’UTR of Fam168a were co‐transfected with negative control (NC) mimics or miR‐337‐3p mimics into C2C12 cells using Lipofectamine 2000 (Invitrogen; Thermo Fisher Scientific Inc.) according to the manufacturer's instructions, with Renilla luciferase plasmid (psi‐CHECK2) as the internal control. Forty‐eight hours after the transfection, the luciferase activity was assessed using a Microplate Reader (GloMax, Promega).

### 2.11 Statistical Analysis

Data were expressed as mean ± standard deviation (SD), and the overall effect of different groups was assessed by one‐way analysis of variance (ANOVA) followed by multiple comparison using Duncan method. Differences between two groups test were analysed using Student's *t*‐test. All the statistical analyses and figure generation were performed using GraphPad Prism version 8.0.0 for Windows (GraphPad Software, San Diego, California USA, www.graphpad.com). The statistical significance was accepted when a *P* value was smaller than 0.05.

## 3 Results

### 3.1 Identification of Potential Dys‐Expressed lncRNAs Related to the Progression of Sarcopenia

Based on a series of analysis of GEO datasets GSE55164 and GSE55163, 49 dys‐expressed miRs (20 down‐regulated and 29 up‐regulated), 29 lncRNAs (four down‐regulated and 25 up‐regulated), and 191 coding genes (27 down‐regulated and 164 up‐regulated) were identified. Then the co‐expression relationships were built using the above lncRNAs and DEGs. Furthermore, the ceRNA network analysis identified eight hub lncRNAs that might be closely related to the progression of sarcopenia (Figure S1), including A430093F15Rik, Gm10782, Gm13405, Gm12259, Gm17203, Gm20635, Gm15337 and Gm2018.

### 3.2 Validation of the Expression Levels of lncRNAs in Musculus Gastrocnemius Tissues of Mice and in C2C12 Cells

**FIGURE 1:**
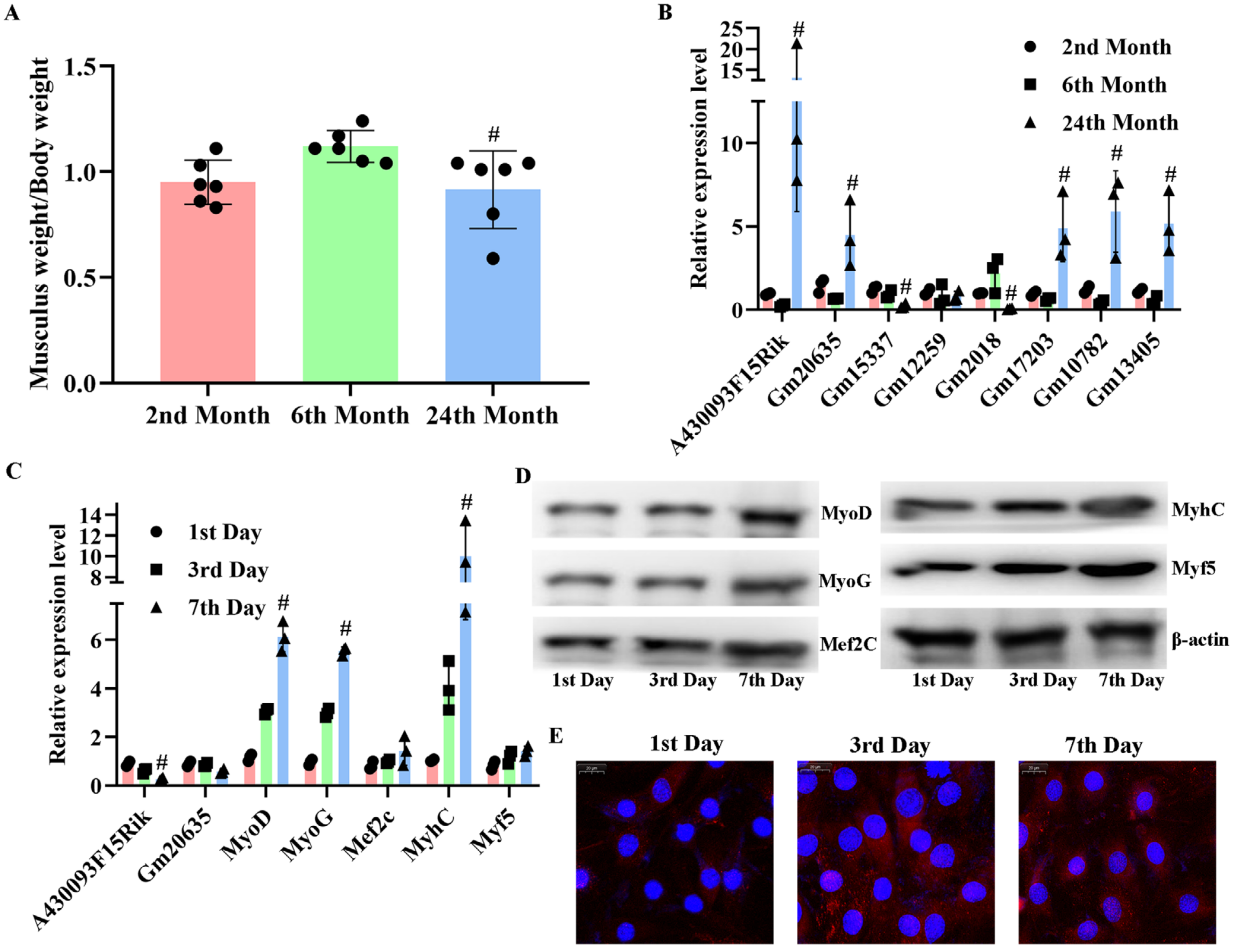
Validation of the expression levels of A430093F15Rik in musculus gastrocnemius tissues of mice and in C2C12 cells. (A) Analysis results of ratio of musculus gastrocnemius weight/body weight with mice ageing. (B) RT‐qPCR detection of different lncRNAs with mice ageing. (C) RT‐qPCR detection of A430093F15Rik and myogenesis‐related indicators with mice ageing. (D) Western blotting detection of myogenesis‐related indicators with mice ageing. (E) FISH detection of A430093F15Rik distribution in C2C12 cells. 2nd Month, mice of two months old. 6th Month, mice of six months old. 24th Month, mice of 24 months old. 1st Day, C2C12 cells of one day culture. 3rd Day, C2C12 cells of three days culture. 7th Day, C2C12 cells of seven days culture. ^#^ *p* < 0.05 vs. 6th Month or 3rd Day.

To confirm the expression levels of the selected lncRNAs, musculus gastrocnemius tissues were collected from mice of two, six and 24 months old. The data showed that the ratio of musculus gastrocnemius weight to body weight was highest at six months and declined with ageing (Figure 1A). Regarding the expression levels of lncRNAs, the levels of A430093F15Rik, Gm10782, Gm13405, Gm12259, Gm17203 and Gm20635 were down‐regulated in adult mice, while the levels of Gm15337 and Gm2018 were up‐regulated. However, the expression pattern of these lncRNAs was reversed in aged mice (Figure 1B), and the differences were all statistically significant (*p* < 0.05). Of the eight lncRNAs, the increase in A430093F15Rik and Gm20635 was higher than that of other lncRNAs (Figure 1B). Subsequent correlation analysis showed that both lncRNAs' levels were positively correlated with the ratio of musculus gastrocnemius weight to body weight, but the *R* ^2^ value (0.73) of A430093F15Rik was much larger than that of Gm20635 (0.47). Moreover, in C2C12 cells, the expression levels of both lncRNAs decreased with cell differentiation as illustrated by the increased expression of myogenesis factors, including MyoD, MyoG and Myhc (Figure 1C,D), with A430093F15Rik showing more obvious decreasing trend in the cytoplasm (Figure 1E). Collectively, A430093F15Rik was selected for subsequent assays.

### 3.3 Target Prediction of A430093F15Rik

**FIGURE 2:**
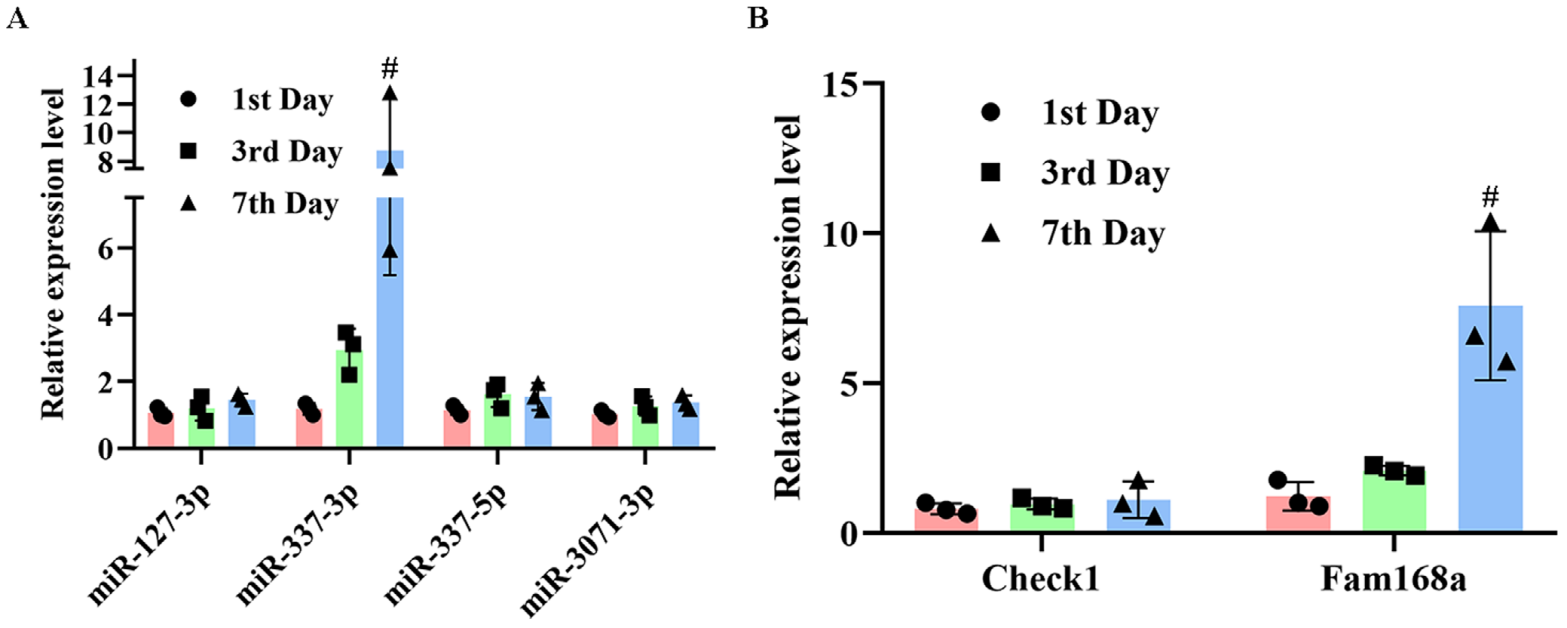
Validation of the downstream effectors of A430093F15Rik in C2C12 cells. (A) RT‐qPCR detection of potential downstream miRs regulated by A430093F15Rik in C2C12 cells. (B) RT‐qPCR detection of the potential downstream miR‐337‐3p in C2C12 cells. 1st Day, C2C12 cells of one day culture. 3rd Day, C2C12 cells of three days culture. 7th Day, C2C12 cells of seven days culture. ^#^ *p* < 0.05 vs. 3rd Day.

Candidate miR interactors of A430093F15Rik were predicted using miRWalk 3.0 and miRanda 3.3a, requiring canonical seed pairing and prediction scores above the default tool thresholds. The potential downstream effectors of A430093F15Rik were identified as follows: including miR‐127‐3p, miR‐3071‐3p, miR‐337‐5p and miR‐337‐3p. Then, the expression status of the four miRs in C2C12 cells was detected. As shown in Figure 2A, the expression levels of all the four miRs increased with cell differentiation, but only the change in miR‐337‐3p was statistically significant (*p* < 0.05). Therefore, the subsequent prediction was performed with miR‐337‐3p, and the results showed that two targets of the miR, including Check1 and Fam168a, were down‐regulated with C2C12 myogenesis (Figure 2B). However, only the change in expression of Fam168a was statistically significant (*p* < 0.05). Collectively, the data suggested that A430093F15Rik might be involved in the initiation of sarcopenia by modulating miR‐337‐3p/Fam168a pathway, and all the subsequent assays were performed focusing on this signalling transduction.

### 3.4 Modulation of A430093F15Rik Influences the Viability and Myogenesis of C2C12 Cells

**FIGURE 3:**
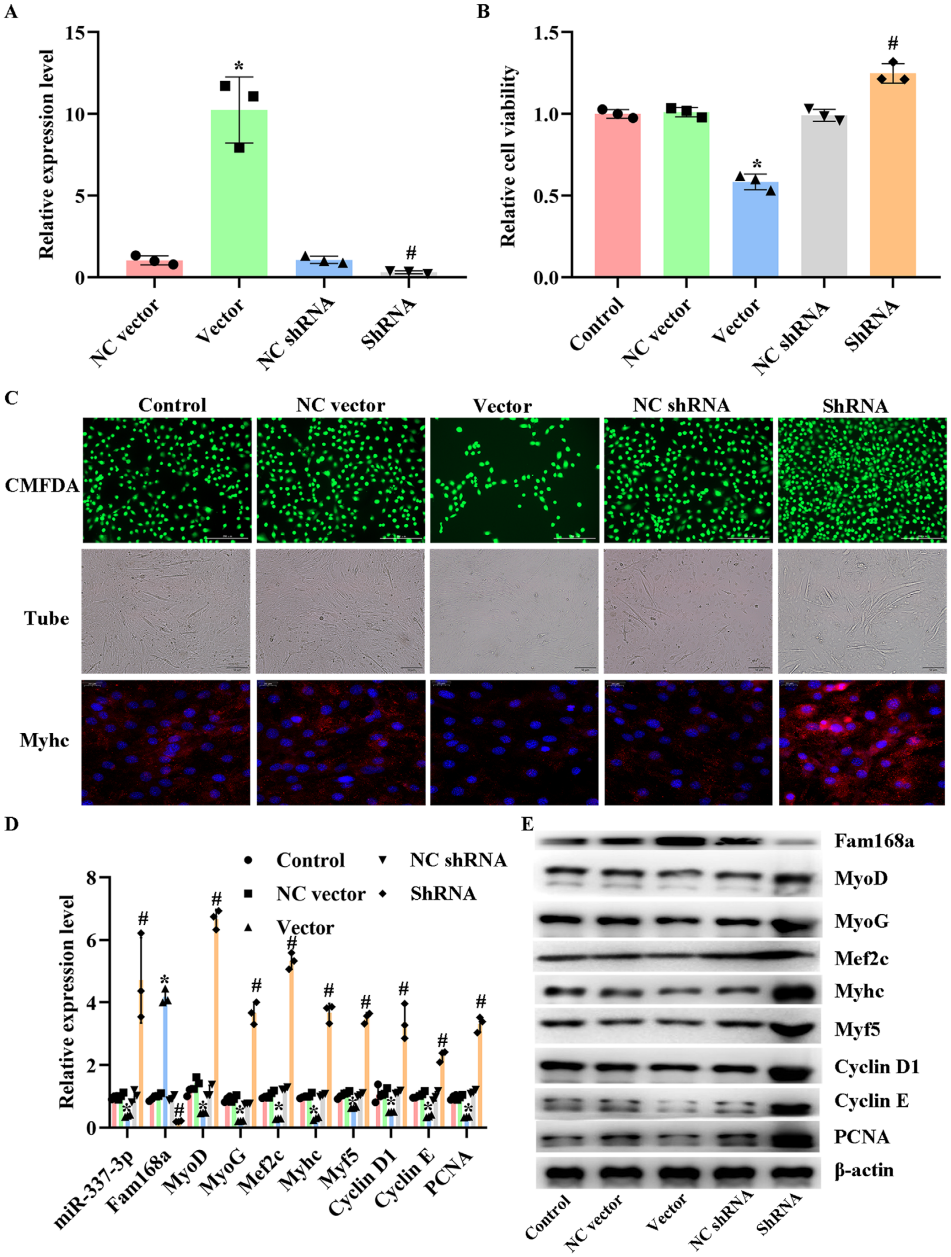
Effects of A430093F15Rik modulation on the viability and myogenesis of C2C12 cells. (A) RT‐qPCR detection of A430093F15Rik levels in C2C12 cells with A430093F15Rik overexpression or inhibition. (B) CCK‐8 assay of cell viability in C2C12 cells with A430093F15Rik overexpression or inhibition. (C) CMFDA staining, tube formation assay, and MyHC IF detection in C2C12 cells with A430093F15Rik overexpression or inhibition. (D) RT‐qPCR detection of miR‐337‐3p, Fam168a and myogenesis‐related indicators in C2C12 cells with A430093F15Rik overexpression or inhibition. (E) Western blotting detection of Fam168a and myogenesis‐related indicators in C2C12 cells with A430093F15Rik overexpression or inhibition. NC vector, C2C12 cells transfected with negative control vector. Vector, C2C12 cells transfected with A430093F15Rik expression vector. NC shRNA, C2C12 cells transfected with non‐targeting shRNA. ShRNA, C2C12 cells transfected with A430093F15Rik shRNA. ^*^ *p* < 0.05 vs. NC vector. ^#^ *p* < 0.05 vs. NC shRNA.

The potential role of A430093F15Rik in the growth and differentiation of myoblasts was further explored by modulating the expression of the lncRNA with a specific expression vector and shRNA (Figure 3A). The overexpression of A430093F15Rik suppressed cell viability (Figures 3B,C), inhibited the formation of myotubes (Figure 3C), and limited myogenesis (Figure 3C–E) in C2C12 cells. However, in cells with A430093F15Rik inhibition, the numbers of myotubes, CMFDA positive cells, MyHC positive cells as well as the expression of myogenesis indicators, including MyoD, MyoG, Mef2c, MyhC, Myf5, Cyclin E, Cyclin D1 and PCNA were all increased (Figure 3B–E). Moreover, the expression of miR‐337‐3p was down‐regulated with A430093F15Rik overexpression, but was up‐regulated with A430093F15Rik inhibition (Figure 3D). Regarding Fam168a, the expression pattern was opposite to that of miR‐337‐3p (Figures 3D,E). The data confirmed the influence of A430093F15Rik on the viability and myogenesis of C2C12 cells.

### 3.5 Validation of the Direct Modulation of the ceRNA Interaction A430093F15Rik/miR‐337‐3p/Fam168a

**FIGURE 4:**
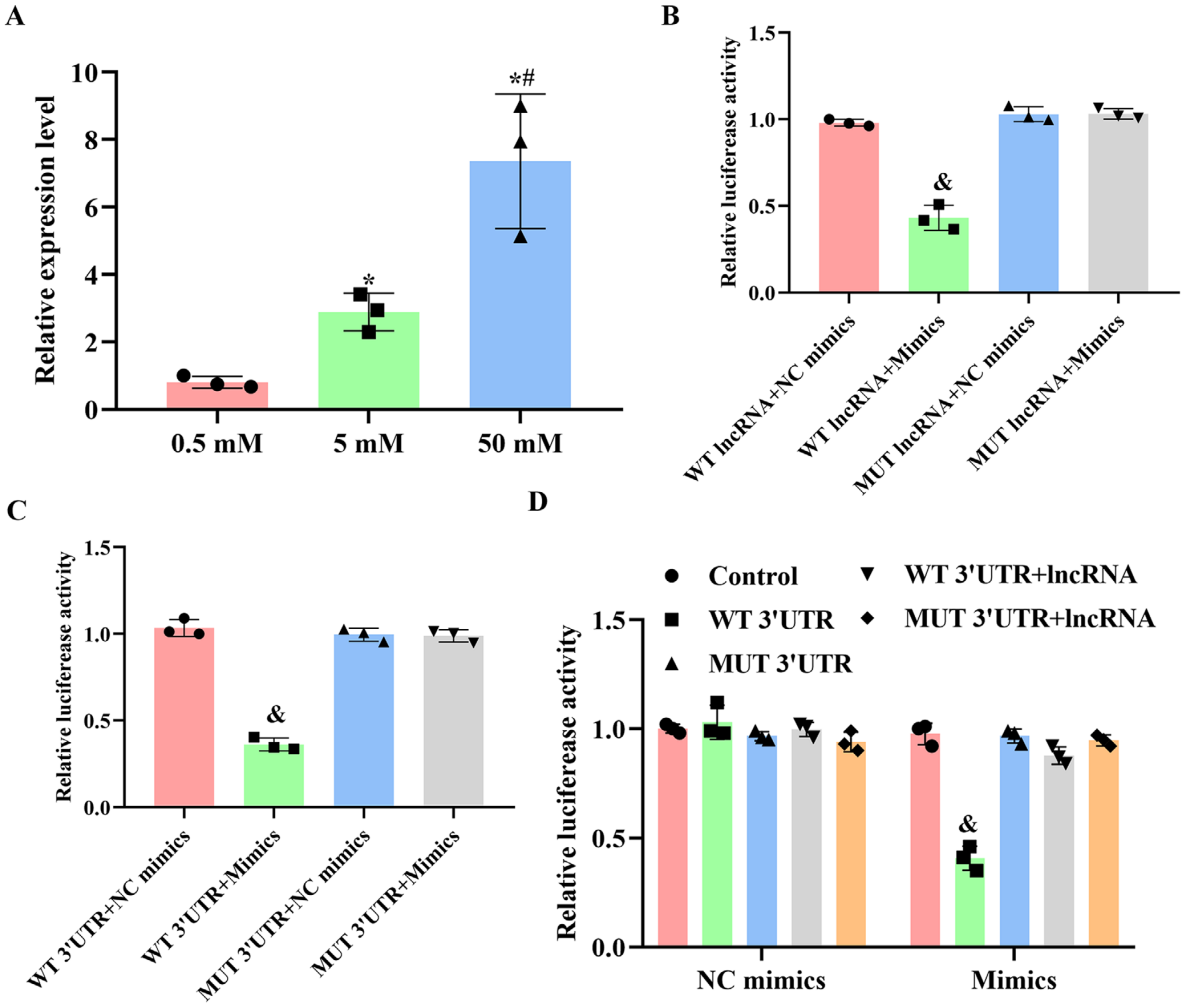
Detection of the direct interaction between A430093F15Rik, miR‐337‐3p and Fam168a in C2C12 cells. (A) RNA pulldown detection of the interaction between A430093F15Rik and miR‐337‐3p in C2C12 cells. (B) Dual luciferase assay detection of the interaction between A430093F15Rik and miR‐337‐3p in C2C12 cells. (C) Dual luciferase assay detection of the interaction between miR‐337‐3p and Fam168a in C2C12 cells. (D) Dual luciferase assay detection of the interaction between miR‐337‐3p and Fam168a in C2C12 cells with A430093F15Rik overexpression. ^*^ *p* < 0.05 vs. 0.5 nM. ^#^ *p* < 0.05 vs. 5 nM. ^&^ *p* < 0.05 vs. WT lncRNA+NC mimics or WT 3’UTR + NC mimics or Control.

The direct interaction and modulation between A430093F15Rik, miR‐337‐3p and Fam168a were validated using RNA‐pulldown and dual luciferase assays. Based on the results of RNA‐pulldown assay, the increased concentration of biotin‐labelled A430093F15Rik contributed to the decreased expression of miR‐337‐3p (Figure 4A), indicating a direct binding between A430093F15Rik and miR‐337‐3p. The conclusion was further supported by the results of the dual luciferase assay using different types of A430093F15Rik vector and miR‐337‐3p mimics: only the co‐transfection of WT A430093F15Rik and miR‐337‐3p mimics suppressed the intensity of fluorescence (Figure 4B), confirming the binding between A430093F15Rik and miR‐337‐3p. Subsequently, the direct modulation of Fam168a by miR‐337‐3p was also verified by a dual luciferase assay: only the co‐transfection of WT Fam168a 3’UTR and miR‐337‐3p mimics suppressed the fluorescence intensity, indicating the direct binding of Fam168a 3’UTR by miR‐337‐3p (Figure 4C). Furthermore, the co‐transfection of WT A430093F15Rik and WT Fam168A 3’UTR blocked the binding of miR‐337‐3p mimics to the 3’UTR sequence (Figure 4D). The data collectively confirmed the existence of the A430093F15Rik/miR‐337‐3p/Fam168a ceRNA interaction in C2C12 cells and indicated that A430093F15Rik increased the expression of Fam168a by competitively binding miR‐337‐3p.

### 3.6 Overexpression of miR‐337‐3p Counteracted the Effects of A430093F15Rik Overexpression in C2C12 Cells

**FIGURE 5:**
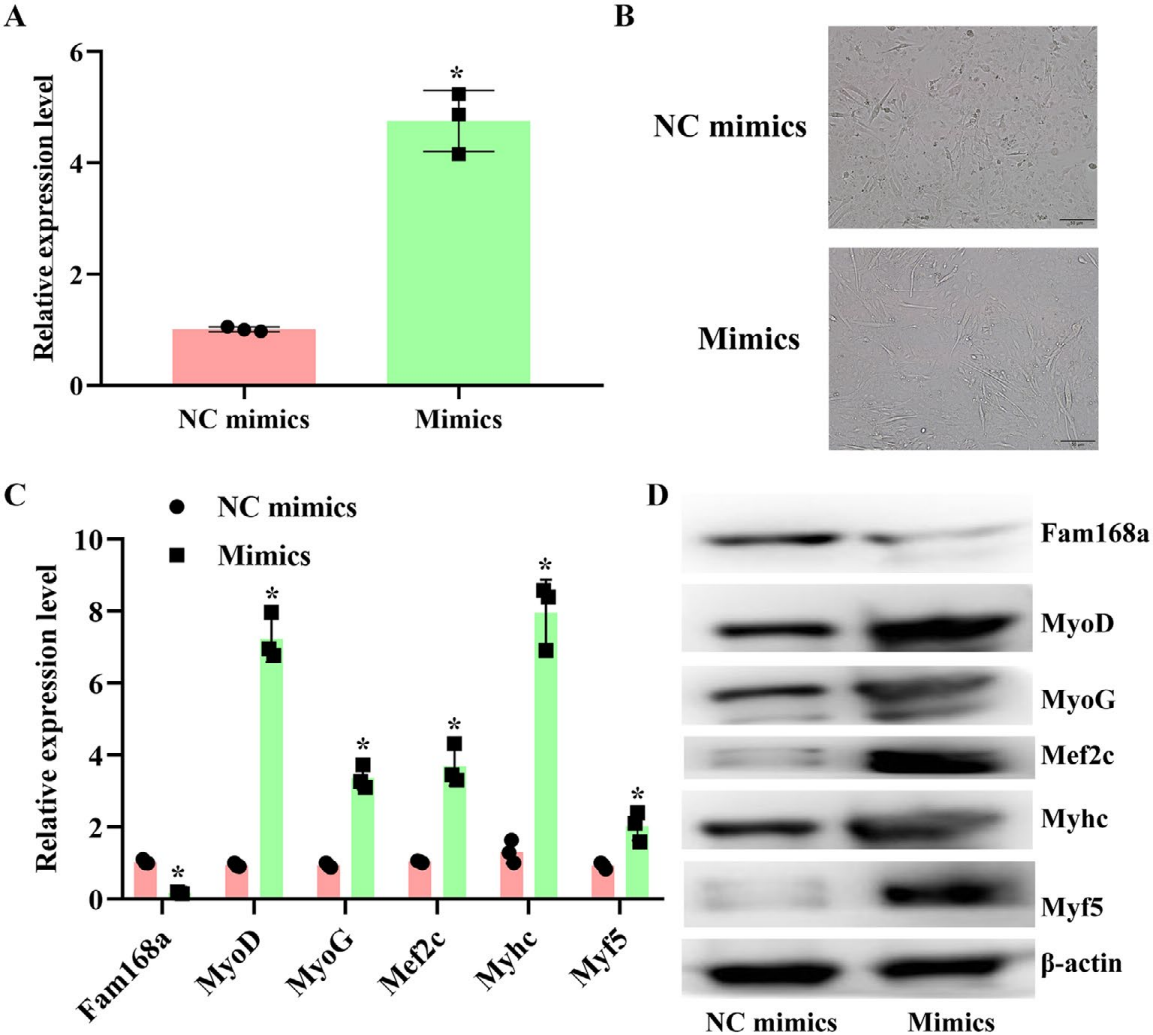
Overexpression of miR‐337‐3p promotes cell proliferation and myogenesis in C2C12 cells. (A) RT‐qPCR detection of miR‐337‐3p levels in C2C12 cells with miR‐337‐3p overexpression. (B) Tube formation assay in C2C12 cells with miR‐337‐3p overexpression. (C) RT‐qPCR detection of Fam168a and myogenesis‐related indicators in C2C12 cells with miR‐337‐3p overexpression. (D) Western blotting detection of Fam168a and myogenesis‐related indicators in C2C12 cells with miR‐337‐3p overexpression. NC mimics, C2C12 cells transfected with non‐targeting mimics. Mimics, C2C12 cells transfected with miR‐337‐3p mimics; ^*^ *p* < 0.05 vs. NC mimics.

**FIGURE 6:**
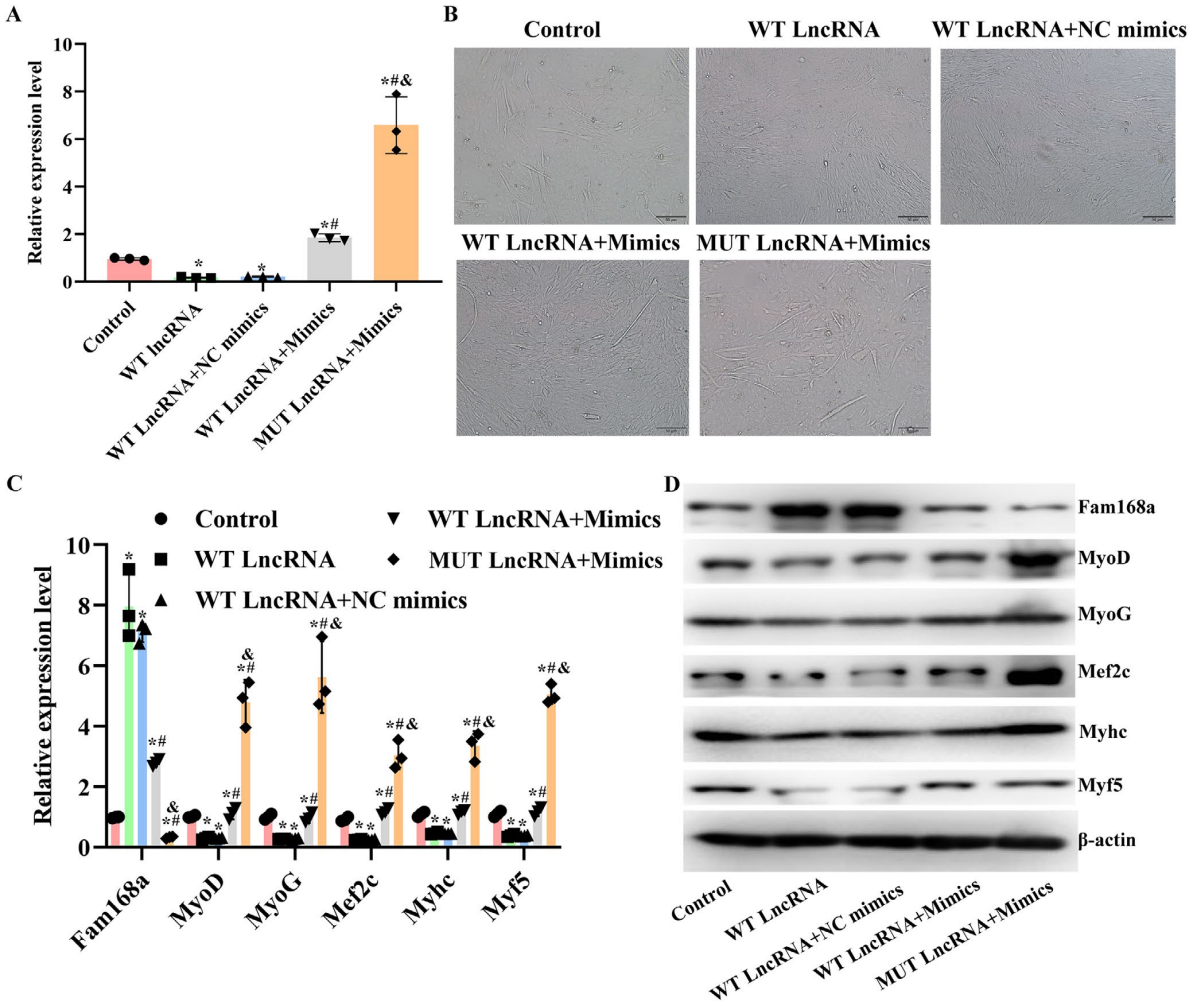
Overexpression of miR‐337‐3p counteracts impairments due to A430093F15Rik in C2C12 cells. (A) RT‐qPCR detection of miR‐337‐3p levels in C2C12 cells with A430093F15Rik overexpression, miR‐337‐3p overexpression or A430093F15Rik mutation. (B) Tube formation assay in C2C12 cells with A430093F15Rik overexpression, miR‐337‐3p overexpression or A430093F15Rik mutation. (C) RT‐qPCR detection of Fam168a and myogenesis‐related indicators in C2C12 cells with A430093F15Rik overexpression, miR‐337‐3p overexpression or A430093F15Rik mutation. (D) Western blotting detection of Fam168a and myogenesis‐related indicators in C2C12 cells with A430093F15Rik overexpression, miR‐337‐3p overexpression or A430093F15Rik mutation. Control, C2C12 cells. WT lncRNA, C2C12 cells transfected with A430093F15Rik vector. WT lncRNA+NC mimics, C2C12 cells transfected with A430093F15Rik vector and non‐targeting mimics. WT lncRNA+Mimics, C2C12 cells transfected with A430093F15Rik vector and miR‐337‐3p mimics. MUT lncRNA+Mimics, C2C12 with A430093F15Rik mutation and transfected with miR‐337‐3p mimics ^*^ *p* < 0.05 vs. Control. ^#^ *p* < 0.05 vs. WT lncRNA+NC mimics. ^&^ *p* < 0.05 vs. WT lncRNA+Mimics.

Following the confirmation of the interaction between A430093F15Rik, miR‐337‐3p and Fam168a, the level of miR‐337‐3p was modulated in C2C12 cells (Figure 5A). The induced level of miR‐337‐3p augmented the tube formation of C2C12 cells and increased the myogenesis potential of the cells while suppressing the level of Fam168a (Figure 5B–D). The beneficial effects of miR‐337‐3p on C2C12 cells were further verified by simultaneously inducing the levels of A430093F15Rik and the miR. It was found that the transfection of miR‐337‐3p mimics counteracted the impairments of A430093F15Rik in C2C12 cells: the suppressed cell tube formation and myogenesis potentials induced by A430093F15Rik were rescued by miR‐337‐3p mimics, and the effects were further strengthened once A430093F15Rik sequence was mutated (Figure 6).

### 3.7 Overexpression of Fam168a Contributed to Sarcopenia Features in C2C12 via Synergistically Functioning With A430093F15Rik

**FIGURE 7:**
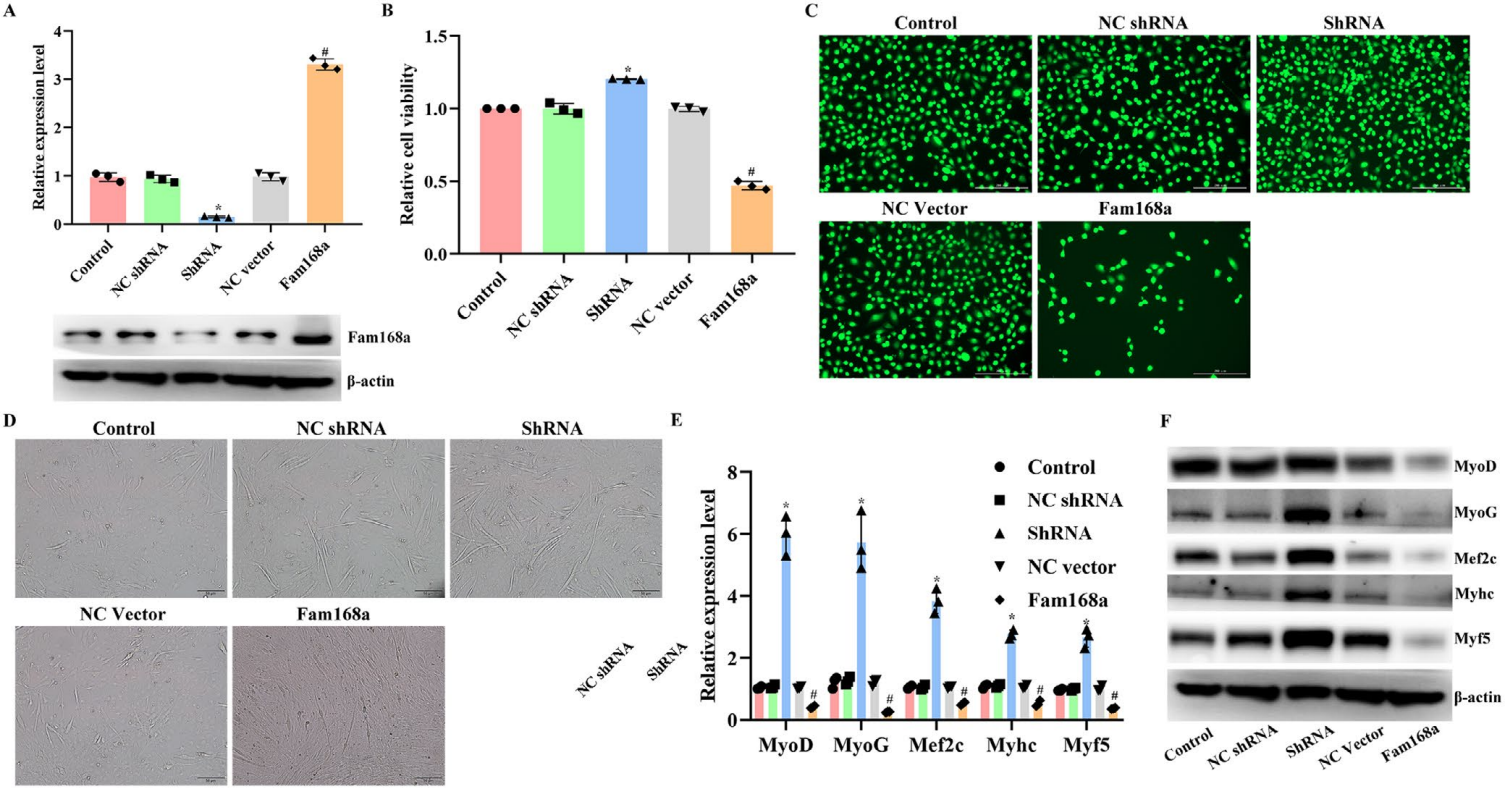
Effects of Fam168a modulation on the viability and myogenesis of C2C12 cells. (A) RT‐qPCR and western blotting detections of Fam168a levels in C2C12 cells with Fam168a overexpression or inhibition. (B) CCK‐8 detection of cell viability in C2C12 cells with Fam168a overexpression or inhibition (relative viability values were normalized to the Control group, set to 1.0). (C) Tube formation assay in C2C12 cells with Fam168a overexpression or inhibition. (D) RT‐qPCR detection of myogenesis‐related indicators in C2C12 cells with Fam168a overexpression or inhibition. (E) Western blotting detection of myogenesis‐related indicators in C2C12 cells with Fam168a overexpression or inhibition. Control, C2C12 cells. NC shRNA, C2C12 cells transfected with non‐targeting shRNA. ShRNA, C2C12 cells transfected with Fam168a shRNA. NC vector, C2C12 cells transfected with negative control vector. Fam168a, C2C12 cells transfected with Fam168a expression vector. ^*^ *p* < 0.05 vs. NC shRNA. ^#^ *p* < 0.05 vs. NC vector.

**FIGURE 8:**
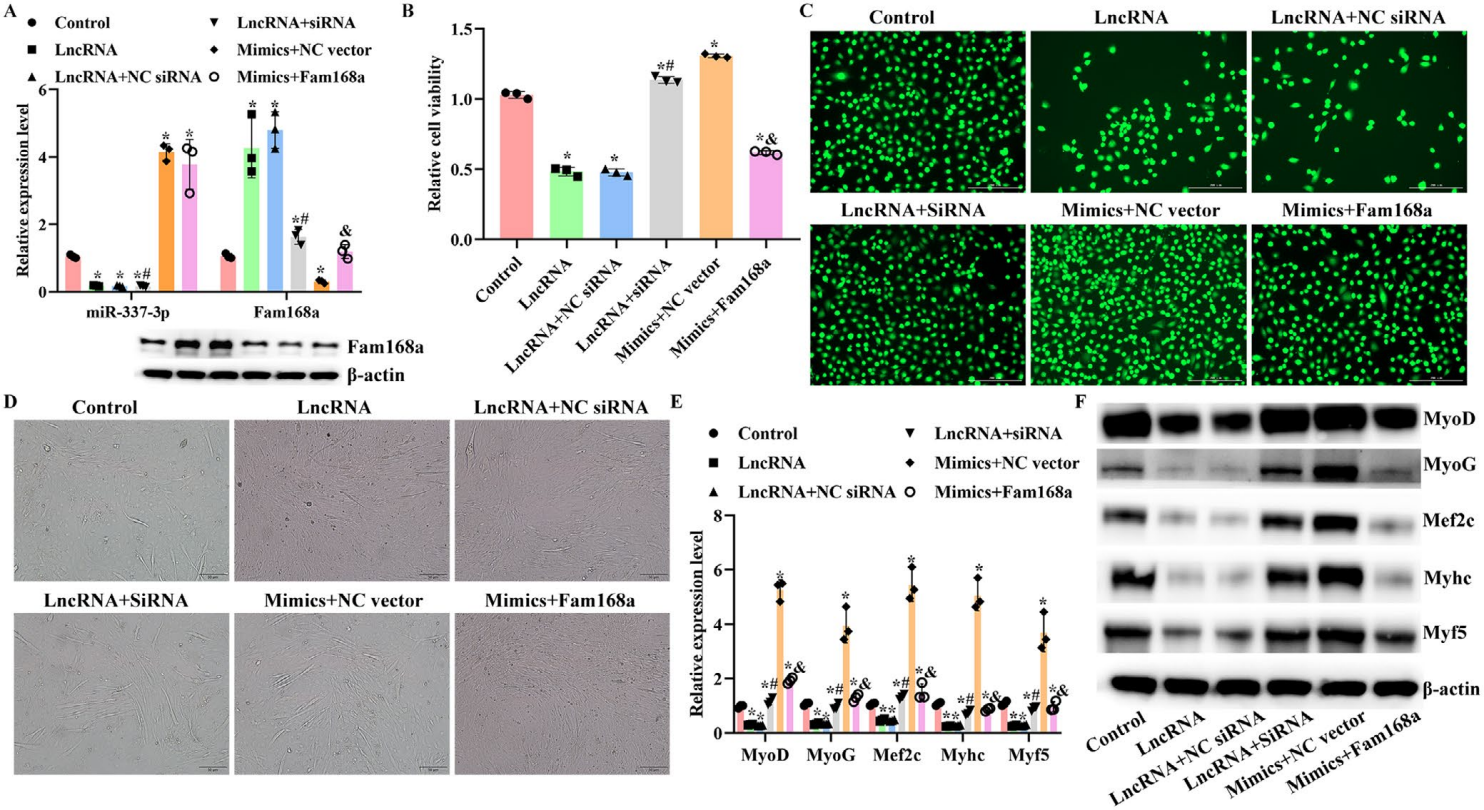
Overexpression of Fam168a contributes to sarcopenia features in C2C12 via synergistically functioning with A430093F15Rik. (A) RT‐qPCR and western blotting detections of miR‐337‐3 and Fam168a levels in C2C12 cells with different modulations of A430093F15Rik, miR‐337‐3p and Fam168a. (B) CCK‐8 detection of cell viability in C2C12 cells with different modulations of A430093F15Rik, miR‐337‐3p and Fam168a (relative viability values were normalized to the Control group, set to 1.0). (C) CMFDA staining in C2C12 cells with different modulations of A430093F15Rik, miR‐337‐3p, and Fam168a. (D) Tube formation assay in C2C12 cells with different modulations of A430093F15Rik, miR‐337‐3p and Fam168a. (E) RT‐qPCR detection of myogenesis‐related indicators in C2C12 cells with different modulations of A430093F15Rik, miR‐337‐3p and Fam168a. (F) Western blotting detection of myogenesis‐related indicators in C2C12 cells with different modulations of A430093F15Rik, miR‐337‐3p and Fam168a. Control, C2C12 cells. LncRNA, C2C12 cells transfected with A430093F15Rik expression vector. LncRNA+NC shRNA, C2C12 cells transfected with A430093F15Rik expression vector and non‐targeting shRNA. LncRNA+NC shRNA, C2C12 cells transfected with A430093F15Rik expression vector and Fam168a shRNA. Mimics+NC vector, C2C12 cells transfected with miR‐337‐3p mimics and negative control vector. Mimics+Fam168a, C2C12 cells transfected with miR‐337‐3p mimics and Fam168a expression vector. ^*^ *p* < 0.05 vs. Control. ^#^ *p* < 0.05 vs. LncRNA+NC shRNA. ^&^ *p* < 0.05 vs. Mimics+NC vector.

The function of Fam168a in the progression of sarcopenia was explored by modulating its expression in C2C12 cells (Figure 7A). The inhibition of Fam168a contributed to the increased tube number and myogenesis potential of C2C12 cells (Figure 7B–F). The subsequent assays with A430093F15Rik and miR‐337‐3p overexpression showed that without the function of Fam168a (Figure 8A), the overexpression of A430093F15Rik could not induce sarcopenia features in C2C12 cells either (Figure 8B–F). Similarly, the overexpression of Fam168a counteracted the effects of miR‐337‐3p mimics (Figure 8B–F). The functional verification confirmed the results of dual luciferase assays and confirmed the regulatory sequence in the A430093F15Rik/miR‐337‐3p/Fam168a axis.

### 3.8 Knockdown of A430093F15Rik Inhibited the Progression of Sarcopenia in Mice

**FIGURE 9:**
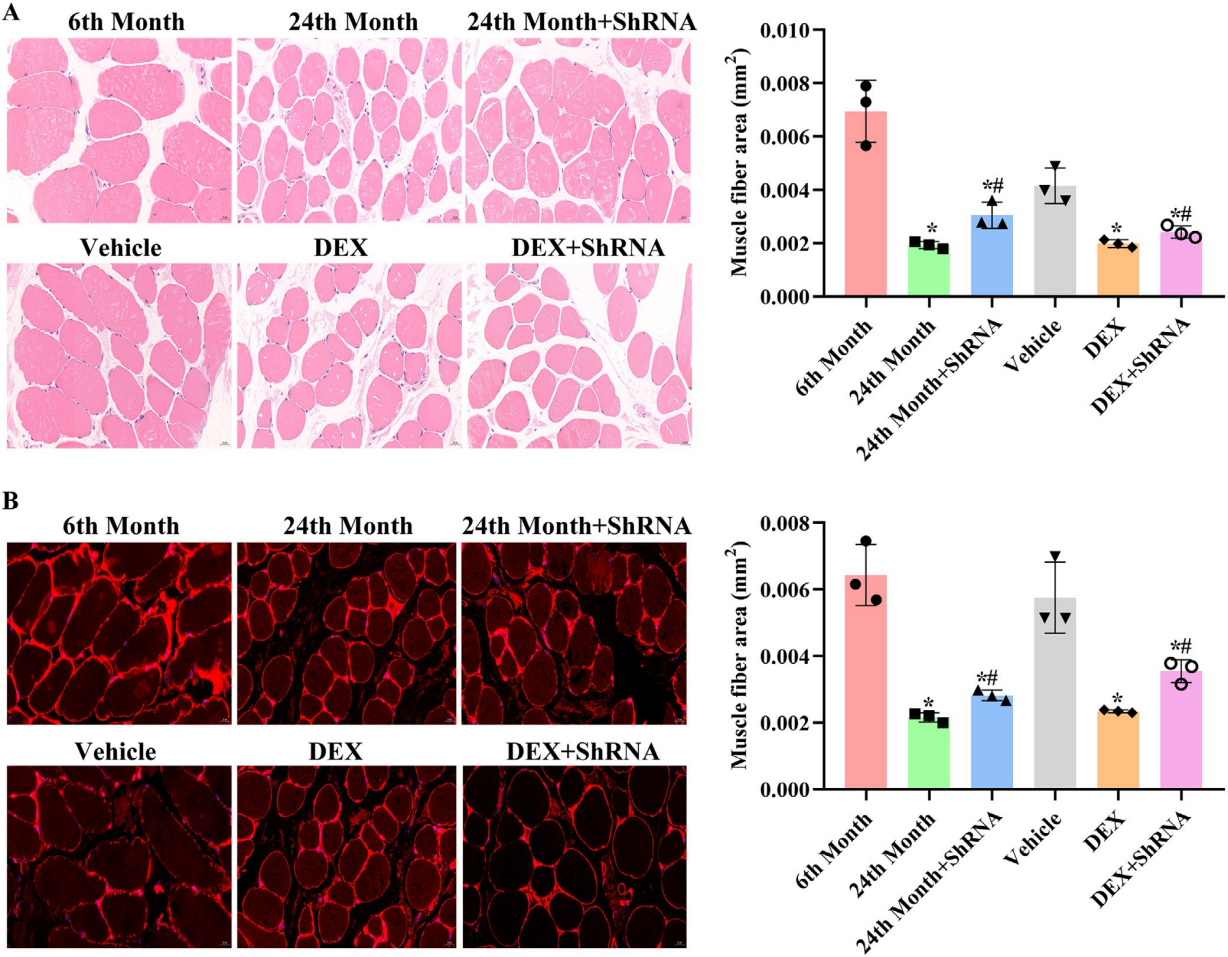
Effects of A430093F15Rik inhibition on the progression of sarcopenia in mice. (A) Representative images and quantitative analysis of H&E staining of averaged area of myofibers in mice. (B) Representative images and quantitative analysis of IF staining of laminin in mice. 6th Month, mice of six months old. 24th Month, mice of 24 months old. 24th Month+shRNA, mice of 24 months old transfected with A430093F15Rik shRNA. Vehicle, six‐month‐old mice subjected to vehicle injection; DEX, six‐month‐old mice subjected to DEX induction (25 μg/g); DEX + shRNA, six‐month‐old mice subjected to DEX induction (25 μg/g) transfected with A430093F15Rik shRNA. ^*^ *p* < 0.05 vs. 6th Month or Vehicle. ^#^ *p* < 0.05 vs. 24th Month or DEX.

The results of in vitro assays were then verified using mice models. As shown in Figure 9, the ageing and DEX treatment substantially decreased the averaged area of myofibers detected by H&E staining (Figure 9A) or by IF staining of laminin (Figure 9B). However, after the inhibition of A430093F15Rik, the development of sarcopenia decelerated: the average area of myofibers increased in aged mice or in mice treated with DEX with A430093F15Rik inhibition compared with mice without A430093F15Rik inhibition (*p* < 0.05). The results derived from mouse model further supported the in vitro data, indicating that A430093F15Rik plays an indispensable role in the development of sarcopenia.

## 4 Discussion

The current study explored the role of the A430093F15Rik/miR‐337‐3p/Fam168a axis in the progression of sarcopenia. The data showed that the levels of A430093F15Rik and Fam168a were up‐regulated, while the level of miR‐337‐3p was down‐regulated with ageing and loss of muscle mass in mouse models. The modulation of the three factors showed that the inhibition of A430093F15Rik and Fam168a, and the induction of miR‐337‐3p, attenuated the symptoms associated with sarcopenia. Moreover, the dual luciferase and RNA‐pulldown assays confirmed the direct interactions between A430093F15Rik and miR‐337‐3p, and miR‐337‐3p and Fam168a, indicating that A430093F15Rik regulates the expression of Fam168a by competitively binding to miR‐337‐3p. The findings outlined in the current study identified novel potential targets for handling sarcopenia and provided valuable information for explaining the mechanism underlying the progression of the disorder.

Loss of muscle strength and mass are two major features of sarcopenia, which has been found to be primarily attributed to genetic alterations. For instance, the dysfunction of mitochondria in myocytes is a primary causative factor leading to muscle degeneration and other aspects of the sarcopenia [26]. Other genes including MTHFR, ACTN3 and NRF2, either synergistically or independently as risk factors for sarcopenia in the elderly population [27]. In addition to the dysregulation of protein coding genes, recent studies also show that non‐coding genes are also involved in the initiation and progression of sarcopenia [28]. For example, miR‐133 has been shown to be down‐regulated in aged C57BL/6 mice and in the C2C12 cell line, and targets of this miR, including MuSk, FOXO3 and SIRT1, are related to muscle degeneration [29]. The inhibition of miR‐133 results in muscle degeneration. The study by Tsai et al. showed that in aged diabetic patients and C2C12 cells, the up‐regulation of let‐7 g‐5p induces muscle atrophy by modulating the levels of TNF‐α and IL‐6 [30]. Regarding the role of lncRNAs, the overexpression of MAR1 can potentially benefit patients with sarcopenia and muscle atrophy [31], while the level of DLEU2 is reported to be up‐regulated in sarcopenic patients [32]. Additionally, the function of lncRNAs is primarily exerted in a ceRNA pattern by sponging specific miRs and then regulating downstream effectors [31, 32]. Therefore, a comprehensive exploration of the lncRNAs involved in the pathogenesis of sarcopenia would contribute to the identification of novel therapeutic targets of the disorder.

The current study firstly employed the GEO database to identify lncRNAs related to sarcopenia via a series of bioinformatics analyses. Based on the analyses and RT‐qPCR validation, lncRNA A430093F15Rik was determined as the novel target related to the progression of sarcopenia. The expression level of the lncRNA in mouse muscle tissues increased with ageing, while in C2C12 cells, the level of A430093F15Rik was negatively related to the levels of myogenesis indicators, thereby indicating the involvement of the lncRNA in the pathogenesis of sarcopenia. Subsequently, the downstream effectors of A430093F15Rik were predicted and the miR‐337‐3p/Fam168a axis was selected. The direct binding of miR‐337‐3p by A430093F15Rik was verified by a dual luciferase assay and an RNA pull assay, suggesting that A430093F15Rik might also exert its function via sponging certain miRs and influencing the expression of downstream effectors. MiR‐337‐3p has been well characterized by its involvement in various cancers. Regarding its role in muscle differentiation, the previous study by Zhang et al. showed that miR‐337‐3p suppresses the proliferation of pulmonary artery smooth muscle by targeting Myo10 [33]. However, in the current study, it was found that miR‐337‐3p played a protective role against sarcopenia‐related features in C2C12 cells.

The potential beneficial effects of miR‐337‐3p on muscles were further verified by exploring the function of Fam168a. The protein is also known as tongue cancer resistance associated protein 1 (TCRP1) and was discovered in studies of multidrug resistance of tongue cancer cells [34]: the protein mediates tumour cell cisplatin resistance and radiotherapy tolerance [34, 35]. Regarding muscle disorders, no previous study has reported its role in the dysfunction of muscle differentiation and function. The current study performed a series of assays by modulating the levels of Fam168a, and the data showed that the inhibition of Fam168a attenuated sarcopenia‐related symptoms, while the overexpression of Fam168a resulted in decreased cell proliferation and myogenesis, thus confirming for the first time the role of Fam168a in muscle disorders such as sarcopenia. Additionally, the interaction between miR‐337‐3p and Fam168a supported the potential ceRNA interaction underlying the function of A430093F15Rik: the lncRNA competitively sponges miR‐337‐3p and inhibits its level, which subsequently induces the expression of Fam168a and increases muscle loss due ageing in mice. The current study also induced sarcopenia in a mouse model using DEX, and data showed that the inhibition of A430093F15Rik in mice attenuated impairments associated with sarcopenia, partially verifying the results of in vitro assays.

Several limitations existed in the current study: first, only male mice were included because of well‐documented sex‐specific differences in sarcopenia progression in C57BL/6J mice [25]. Older males display a higher incidence of sarcopenia and clearer molecular alterations such as reduced mitochondrial biogenesis and impaired oxidative capacity, whereas age‐matched females show fewer confirmed cases and lack consistent changes in major pathways. However, we recognize that excluding females limits generalizability, and future studies will incorporate sex‐stratified analyses. Second, A430093F15Rik is up‐regulated during ageing in mice, genomic comparison indicates that it lacks a direct human ortholog. This observation is consistent with prior literature noting that lncRNAs involved in skeletal muscle development and sarcopenia often display species‐specific expression and limited cross‐species conservation. Epigenetic reviews of sarcopenia have repeatedly highlighted that many regulatory lncRNAs are mouse‐specific despite acting on conserved downstream pathways such as mitochondrial regulation, inflammatory signalling and myogenic transcription [28, 36, 37, 38]. Third, only three‐age groups were used (2, 6 and 24 months). It is well established that they represented established physiological stages in C57BL/6J mice and allowed clear discrimination of young, adult and aged muscle states. However, they did not capture the nonlinear trajectory of sarcopenia development, and additional intermediate ages such as 12 or 18 months would better resolve the temporal pattern of ncRNA alterations and will be included in future longitudinal designs. Fourth, while H&E and laminin IF staining showed consistent overall trends, minor discrepancies in absolute fibre‐area values likely reflect differences in staining contrast and segmentation algorithms. Functional muscle testing (e.g., grip strength, treadmill endurance) was not included and represents an important future improvement to confirm the physiological relevance.

In conclusion, the current study identified the novel lncRNA A430093F15Rik as a modulator involved in the progression of sarcopenia. The lncRNA competitively sponges miR‐337‐3p to regulate the expression of Fam168a in a ceRNA pattern. The A430093F15Rik/miR‐337‐3p‐Fam168a axis was first reported to participate in the initiation and development of sarcopenia, especially for miR‐337‐3p and Fam168a, the functions of which have been previously described in other systems. The findings outlined in the current study not only provide a new explanation for the pathogenesis of sarcopenia but also may be employed for the future handling of the disorder. Based on the data in the current study, the future exploration of agents targeting A430093F15Rik/miR‐337‐3p‐Fam168a axis will be performed to promote the management of sarcopenia.

## Author Contributions

**Qing Fang:** conceptualization (equal). **Jianwei Huang:** conceptualization (equal). **Dongping Huang:** conceptualization (equal). **Zhenyi Jia:** conceptualization (equal).

## Funding

The study was supported by the Public Health Research Project of Putuo District in Shanghai (ptgw202202) & Project for Clinical‐featured Specific Disease of Putuo District Health Commission in Shanghai (2023tszb02).

## Ethics Statement

All procedures were conducted in accordance with National Research Council's Guide for the Care and Use of Laboratory Animals, and the protocol was approved by the Institutional Committee for the Care and Use of Laboratory Animals (DWSY2023‐0178, Shanghai Sixth People's Hospital, China).

## Conflicts of Interest

The authors declare no conflicts of interest.

## Supporting information


**Figure S1:** Results of ceRNA network analysis.

**Table S1:** Primer information.

## Data Availability

The datasets used and/or analysed during the current study are available from the corresponding author on reasonable request.
