# Supplementary figures and images for: Inhibited Differentiation and Growth of Myocyte Associated With Sarcopenia: The Key Role of the lncRNA A430093F15Rik/microRNA‐337‐3p/Fam168a Pathway
Source: J Cell Mol Med. 2026 Apr 16;30(8):e71133. doi: 10.1111/jcmm.71133 (PMC13085175; doi:10.1111/jcmm.71133)

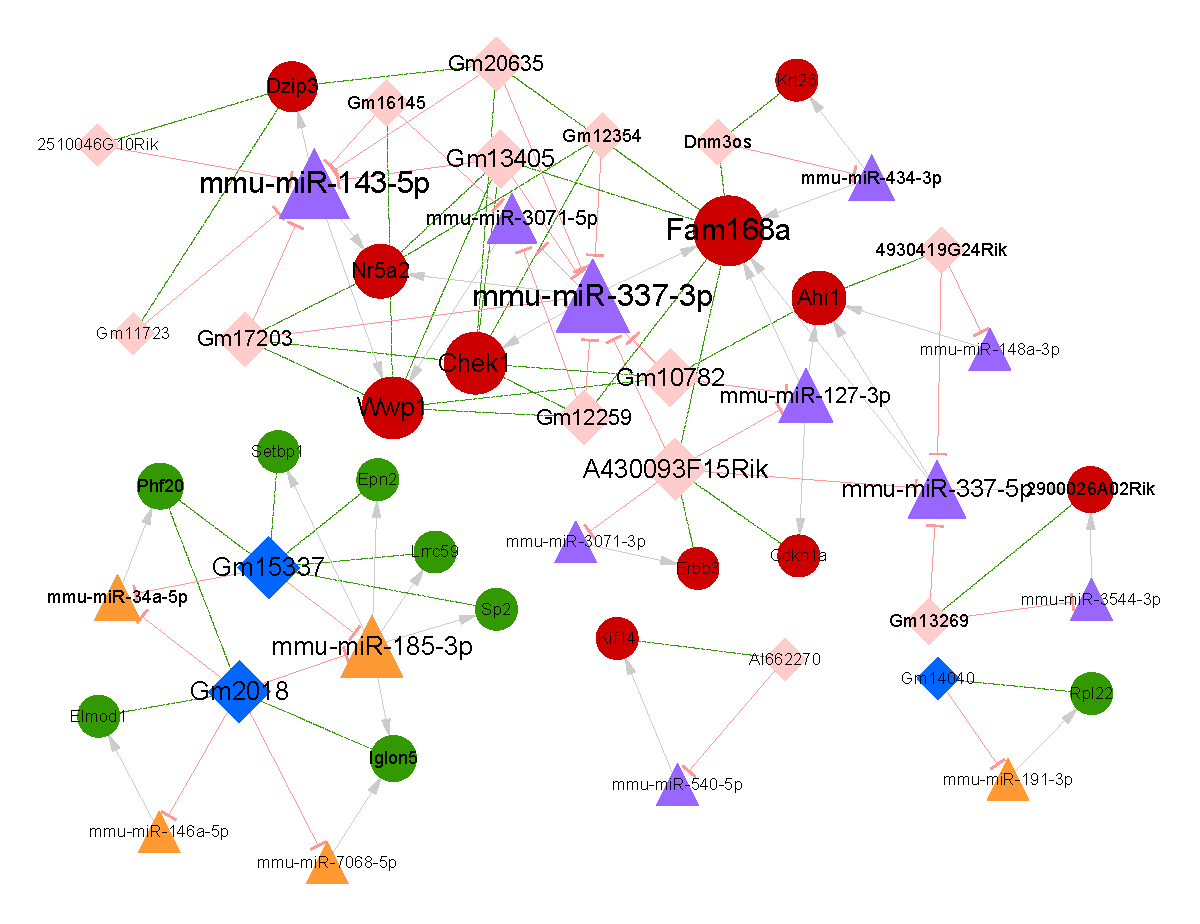

Supplement: Supplementary file 1 — Figure S1: Results of ceRNA network analysis. [file JCMM-30-e71133-s001.tiff]
