# Supplementary material for: Inhibited Differentiation and Growth of Myocyte Associated With Sarcopenia: The Key Role of the lncRNA A430093F15Rik/microRNA‐337‐3p/Fam168a Pathway
Source: J Cell Mol Med. 2026 Apr 16;30(8):e71133. doi: 10.1111/jcmm.71133 (PMC13085175; doi:10.1111/jcmm.71133)
Supplement: Supplementary file 2 — Table S1: Primer information. [file JCMM-30-e71133-s002.docx]

**Table S1 Primer information**

| Gene | Direction | Sequence (5’-3’) |
| --- | --- | --- |
| GAPDH | F | CATCACTGCCACCCAGAAGACTG |
|  | R | ATGCCAGTGAGCTTCCCGTTCAG |
| MyoD | F | GCACTACAGTGGCGACTCAGAT |
|  | R | TAGTAGGCGGTGTCGTAGCCAT |
| MyoG | F | CCATCCAGTACATTGAGCGCCT |
|  | R | CTGTGGGAGTTGCATTCACTGG |
| Mef2c | F | GTGGTTTCCGTAGCAACTCCTAC |
|  | R | GGCAGTGTTGAAGCCAGACAGA |
| Myf5 | F | GGTGGAGAACTATTACAGCCTGC |
|  | R | ACAGTAGATGCTGTCAAAGCTGC |
| MyhC | F | GCTGGAAGATGAGTGCTCAGAG |
|  | R | CCAGCCATCTCCTCTGTTAGGT |
| Cyclin E | F | AAGCCCTCTGACCATTGTGTCC |
|  | R | CTAAGCAGCCAACATCCAGGAC |
| Cyclin D | F | GCAGAAGGAGATTGTGCCATCC |
|  | R | AGGAAGCGGTCCAGGTAGTTCA |
| PCNA | F | CAAGTGGAGAGCTTGGCAATGG |
|  | R | GCAAACGTTAGGTGAACAGGCTC |
| A430093F15Rik | F | TATGACATTGGACACGAAGTATG |
|  | R | GTCTCCAGCACTGACTGACAGTA |
| mmu-miR-127-3p | F | CGTCGGATCCGTCTGAGC |
|  | R | AGTGCAGGGTCCGAGGTATT |
| mmu-miR-337-3p | F | GCGCGTCAGCTCCTATATGAT |
|  | R | AGTGCAGGGTCCGAGGTATT |
| mmu-miR-337-5p | F | GCGGCGTCATGCAGGAG |
|  | R | AGTGCAGGGTCCGAGGTATT |
| mmu-miR-3071-3p | F | GCGCGATCATCAAAACAAATG |
|  | R | AGTGCAGGGTCCGAGGTATT |
| U6 | F | CTCGCTTCGGCAGCACAT |
|  | R | TTTGCGTGTCATCCTTGCG |
